# Towards a Molecular Understanding of the Fanconi Anemia Core Complex

**DOI:** 10.1155/2012/926787

**Published:** 2012-05-22

**Authors:** Charlotte Hodson, Helen Walden

**Affiliations:** Protein Structure and Function Laboratory, Lincoln's Inn Fields Laboratories, London Research Institute, Cancer Research UK, 44 Lincoln's Inn Fields, London WC2A 3LY, UK

## Abstract

Fanconi Anemia (FA) is a genetic disorder characterized by the inability of patient cells to repair DNA damage caused by interstrand crosslinking agents. There are currently 14 verified FA genes, where mutation of any single gene prevents repair of DNA interstrand crosslinks (ICLs). The accumulation of ICL damage results in genome instability and patients having a high predisposition to cancers. The key event of the FA pathway is dependent on an eight-protein core complex (CC), required for the monoubiquitination of each member of the FANCD2-FANCI complex. Interestingly, the majority of patient mutations reside in the CC. The molecular mechanisms underlying the requirement for such a large complex to carry out a monoubiquitination event remain a mystery. This paper documents the extensive efforts of researchers so far to understand the molecular roles of the CC proteins with regard to its main function in the FA pathway, the monoubiquitination of FANCD2 and FANCI.

## 1. Introduction

**Figure 1:**
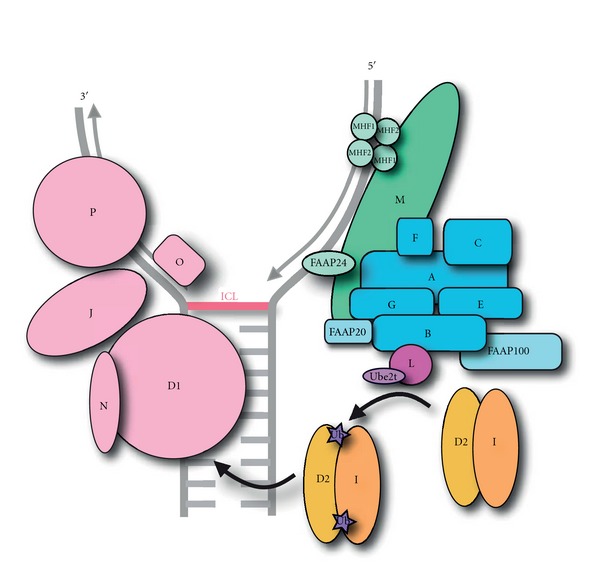
The Fanconi Anemia Pathway. A model of the Fanconi Anemia Pathway at a stalled DNA (grey) replication fork, caused by an interstrand crosslink (ICL). FANCM and its associated genes are coloured green, which assemble on DNA at the stalled replication fork. The other CC proteins are represented by blue with FANCL as the E3 ligase of the CC represented by mauve. The substrates for ubiquitination FANCD2 and FANCI are coloured gold and peach, respectively, with their associated ubiquitins represented by purple stars. The DNA repair machinery is coloured pink.

Fanconi Anemia (FA) patients present a variety of symptoms including skeletal and developmental defects, bone marrow failure, and a high predisposition to cancer [1]. The predisposition to cancer is attributed to the FA pathway being involved in DNA damage repair, particularly interstrand crosslinks (ICLs). FA patients are highly susceptible to crosslinking agents such as mitomycin C (MMC) and cisplatin. Such treatment results in chromosome abnormalities, and sensitivity to these agents is used as a diagnostic tool for FA [2]. Currently there are 14 verified FA genes [3–22], with a possible additional gene FANCO/Rad51C [23, 24], that make up the FA pathway. Mutations in any of the 15 genes results in the loss of ICL repair. The key event of the FA pathway is the monoubiquitination of FANCD2 and FANCI [4–7], which triggers the downstream factors, FANCP/SLX4, FANCD1/BRCA2, FANCJ/BRIP1, FANCN/PALB2 [9, 10, 17–22] to repair DNA damage (Figure 1). Only one protein to date has been shown to have E3 ubiquitin ligase activity, FANCL [8]. FANCL is a member of the Fanconi Anemia Core Complex (CC), consisting of 7 other FA proteins: FANCA, FANCB, FANCC, FANCE, FANCF, FANCG, and FANCM (Figure 1) [3, 25–28]. Additionally, there are the Fanconi Anemia Associated Proteins (FAAPs), which are not yet found mutated in patients but form part of the CC: FAAP100, FAAP24, and most recently FAAP20 [29–33]. MHF1 and MHF2 (also known as FAAP16 and FAAP10) have also been implicated in the FA pathway through their association with FANCM [34, 35]. Approximately 90% of patient mutations reside in the CC, most of which are found in FANCA (60%) (Table 1) [36]. Importantly a single mutation in any of the 8 genes that make up the CC prevents the key monoubiquitination event from occurring. Extensive research efforts have been made to understand the role of the individual CC proteins and the requirement of all CC proteins for the monoubiquitination event. This paper outlines our current understanding of the molecular interactions within the core complex and highlights key remaining questions for a full molecular understanding of the CC.

## 2. E3 Ligase Function of the CC

Patient mutations in any member of a CC protein result in the loss of the critical monoubiquitination of the FANCI/FANCD2 complex. All CC proteins appear to be required for this event *in vivo;* therefore, historically the CC has been regarded as a multisubunit E3 ligase. Multisubunit E3 ligases such as the Cullin-RING ligases (CRLs) and the Anaphase Promoting Complex (APC) are well understood at the molecular level, with their modularity essential for function. 

**Figure 2:**
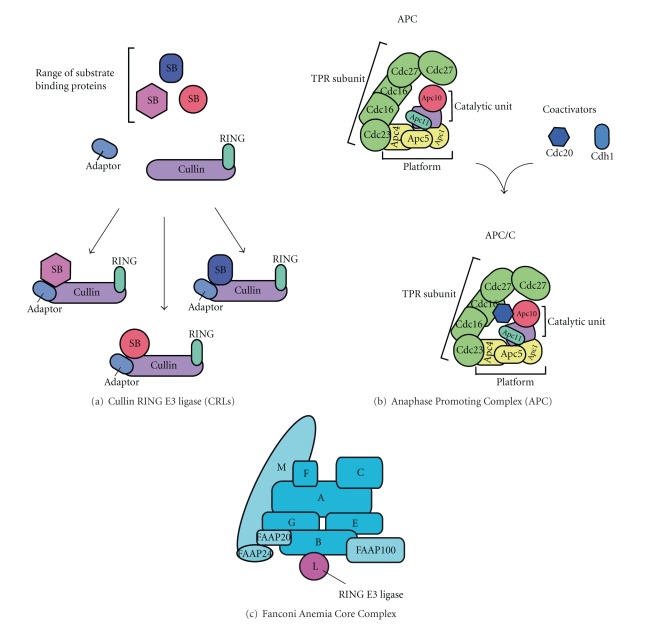
Models of multisubunit E3 ligases. (a) A model of the proteins that make a Cullin-RING E3 ligase (CRL). The Cullin protein (lilac) acts as scaffold and binds the RING domain (cyan) required for E2 binding and the substrate binding proteins (pink, red, blue) via an adaptor. The variety of substrate binding proteins allows the CRLs flexibility in binding a range of substrates. (b) A model of the Anaphase Promoting Complex (APC). The catalytic core consists of a Cullin repeat protein Apc2 (lilac), which acts a scaffold for the RING protein, Apc11 (cyan), and the substrate binding protein Apc10 (red). For substrate recognition the APC also binds coactivators (dark blue). The APC also consists of a TPR subcomplex (green) and a platform (yellow) which orient the catalytic unit and aid binding to the co-activators. The range of subunits allows a variety of substrates to be recognised. (c) A model of the Fanconi Anemia Core Complex. The catalytic activity resides in one protein FANCL (mauve). The rest of the CC proteins are coloured blue, with light blue representing proteins associated with the CC.

The CRLs consist of a Cullin scaffold protein, which associates with either Rbx1 or Rbx2 RING proteins, the subunit responsible for binding the E2 carrying the activated ubiquitin moiety [37]. The Cullin and RING therefore form the catalytic unit of the CRL. In order for a substrate to become ubiquitinated, it must be recognised by the CRL. This is achieved by the substrate receptor proteins, which associate through an adaptor protein onto the Cullin scaffold, forming the complete CRL (Figure 2(a)) [37]. A plethora of different substrate recognition proteins for a single Cullin achieves flexibility within the CRLs to target a repertoire of substrates. 

The APC also targets a variety of substrates to control cell cycle progression from metaphase to anaphase. Similarly to the CRLs the APC comprises of a Cullin repeat protein Apc2, which binds the RING protein Apc11, and also Apc10 involved in substrate association. Together, these 3 subunits form the catalytic unit. However, in contrast to CRLs the APC contains an additional 10 proteins (Figure 2(b)). Apc9 and 13 and Cdc26 are structural stabilizers, whereas Apc1, 4, and 5 form a scaffold platform for the catalytic unit [38]. The scaffold platform along with the tetratricopeptide repeat (TPR) proteins Cdc23, Cdc27, Cdc16, and structural stabilizer Cdc26 forms the TPR subcomplex, orientating the catalytic unit for its association with coactivators, Cdc20 and Cdh1 [38]. The co-activators are required along with Apc10 for substrate recognition [38–40]. In common with the CRLs, this ensemble allows flexibility and diversity in substrate recognition. 

By contrast, the CC has one subunit with E3 ligase activity, FANCL [8] (Figure 2(c)), shown to be the only subunit of the CC required for FANCD2 monoubiquitination *in vitro* [41]. FANCL is a RING E3 ligase [42], which binds the E2 of the FA pathway, Ube2t [41, 43] in a canonical fashion through its RING domain [43, 44]. Earlier *in vitro *and *in vivo* work indicated a FANCE-FANCD2 interaction [45] and a series of yeast and mammalian 2-hybrid studies further support this interaction [46–48]. The interaction of FANCD2 with a CC component prompted the idea that FANCE may bring the substrates FANCD2 and FANCI into close proximity of FANCL for their subsequent monoubiquitination. As with other multisubunit E3 ligases, this would leave FANCL as the “catalytic” subunit, indirectly ubiquitinating substrates through its interaction with E2. However, not only is FANCL sufficient *in vitro* for the monoubiquitination event [41], but also has been shown to interact directly with both FANCD2 and FANCI *in vitro* [42, 44], and with FANCD2 in cells [49]. Although mutations in other CC proteins result in a loss of the monoubiquitination event, these more recent findings suggest FANCL possesses all the requirements to be able to carry out the monoubiquitination, unlike the multisubunit E3 ligases. 

## 3. Protein-Protein Interactions Required for CC Stability

Although the monoubiquitination event *in vitro* requires only FANCL, it is clear from patient mutations that all members of the CC are required *in vivo*. The reasons for this are not clear, although numerous groups have shown the CC proteins interact with one another and are required to form a stable CC. Part of the challenge of gaining a molecular understanding of the core complex lies in the lack of obvious domain structures from primary sequences in any of the proteins except FANCL and FANCM. This section will describe the efforts to understand the molecular biology of the core complex to date. 

### 3.1. FANCG-FANCA CC Interactions

**Figure 3:**
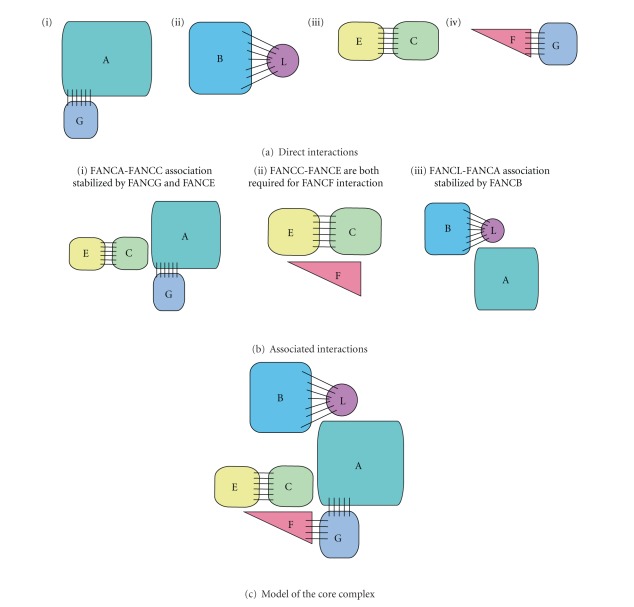
A model of CC interactions. (a) Models of the CC proteins that directly interact with one another, represented by black lines. Yeast and mammalian 2-hybrid and *in vitro* and translational studies have shown these interactions. (b) Associated CC interactions as shown by mammalian and yeast 3-hybrid experiments. (c) A model of how all the CC proteins interact to from the full CC, the requirement for the monoubiquitination event.

FANCG and FANCA have been shown to interact directly and indirectly through yeast 2-hybrid, co-immunoprecipitations (co-IPs), cell-based studies, and *in vitro* translational (IVT) work [25, 26, 46, 50–55] (Figure 3). Co-IPS and IVT studies suggested the N-terminal 300 residues of FANCA bind FANCG [50, 51]. This region has been further narrowed down to the first 40 amino acids by a yeast 2-hybrid assay and the first 37 amino acids by a co-IP study [25, 46], with a requirement for basic amino acids within this region for the interaction [25]. IVT studies indicate residues 18–29 of FANCA are sufficient for a FANCG interaction, specifically Arginine 18, Arginine 19, and Leucine 25 [50]. Studies aimed at identifying the regions of FANCG involved in a FANCA interaction are more conflicting. Hussain et al. [55] reported via yeast 2-hybrid analysis that the predicted TPR motifs 5 and 6 of FANCG, which reside in the C-terminal 170 residues, were required for the interaction. Consistent with this finding, IVT assays also revealed two FANCA binding regions in the C-terminal 222 residues of FANCG: one encompassing the same predicted TPR motifs 5 and 6, and the second residing in the last 37 residues of FANCG [50]. However, in contrast, a yeast 2-hybrid study found the C-terminal 142 amino acids of FANCG to be dispensable for interaction with FANCA [46]. To add to the complexity, other studies report a requirement for additional regions throughout FANCG for a FANCA interaction [52, 54, 56] with Wilson et al.'s [56] *in vivo* study indicating several regions, TPR motifs 1, 2, 5, and 6 throughout FANCG are required. 

FANCG null lymphoblasts have a defect in FANCA nuclear accumulation, which can be rescued by the addition of FANCG, suggesting that FANCG plays a role in the subcellular localization of FANCA [26]. Indeed, the FANCG interaction with FANCA appears to promote FANCA nuclear accumulation [26]. However, a thorough analysis of multiple FANCA patient mutations suggests that FANCG binds FANCA even when the nuclear localisation of FANCA is lost [57]. The FANCA extreme N-terminus contains a nuclear localisation signal (NLS) [25, 58]. FANCA patient mutations are varied and account for 60% of all FA cases (Table 1) and predominantly result in loss of FANCA nuclear accumulation [57]. It appears likely that a combination of the NLS on FANCA and FANCG-binding stabilise, and supports the nuclear subcellular localisation of the core complex. 

These studies all indicate a likely physical interaction between FANCG, and FANCA, but the molecular details have yet to be fully resolved. 

### 3.2. FANCF CC Interactions

FANCF has been implicated in the physical stability of the majority of other CC proteins, FANCC, FANCE, FANCG and FANCA by several groups [27, 28, 59, 60]. X-ray crystallographic analysis of a C-terminal portion of FANCF (residues 156–357) revealed an architecture of helical repeats [60], similar to those found in scaffolding proteins. Structure-based mutations were then generated for use in mammalian co-IP assays. Using both point mutations L209R and F251R and a hydrophobic patch mutation Y287A/L289A/F339A/V341A/L344A, Kowal and coworkers [60] verified and provided the molecular details of the FANCF associations with FANCA and FANCC reported from earlier co-IP studies [27]. In addition, a further association with FANCE was identified [60]. In contrast to the structure-based analysis, Léveillé et al. [59] use coimmunoprecipitation from cultured lymphoblasts and report the requirement of the last 31 amino acids (343–374) of FANCF for a FANCG and FANCA interaction and additionally report that the first 15 N-terminal amino acids are required for a FANCE and FANCC interaction. 

Yeast and mammalian 3-hybrids revealed that a FANCC-FANCE interaction was required for a direct interaction with FANCF [59, 61]. In accordance with their co-IP studies, Léveillé et al. [59] show residues Leu5/Leu8/Leu15 are required for a FANCC-FANCE interaction in their mammalian 3-hybrid assay and report two additional regions, Arg10/Phe11/Arg47/Phe48 and Ser18/Ser19/Thr20/Thr21, also required for this interaction [59]. Whilst these findings are not necessarily incompatible, the molecular details of the interactions between FANCF and FANCG, FANCA and FANCE are still unresolved. 

The FANCA-FANCF interaction is mediated by FANCG, from yeast 3-hybrid analyses [46]; conversely co-IP studies indicate a FANCA-FANCG interaction is stabilized by FANCF [27]. Both these observations are supported by yeast 2-hybrid experiments that show a direct interaction of FANCG with FANCF [28, 46, 55, 59]. Gordon and Buchwald [46] show that this interaction resides in the last 131 C-terminal residues of FANCF, and Léveillé et al. [59] narrow this down to the last 40 amino acids in their yeast 2-hybrid assay. Several groups have attempted to map the region of FANCG responsible for a FANCF interaction by yeast 2-hybrid analysis, all of which conclude that several sites are required throughout the full amino acid sequence of FANCG [46, 52, 55]. 

Importantly, structure-guided mutagenesis of FANCF increased MMC sensitivity, thereby directly showing FANCF interactions are critical [60]. Although there are conflicting results regarding FANCF associations with other CC members, numerous studies all support the role for FANCF in coordinating and stabilizing other CC proteins, as seen for FANCG. 

### 3.3. FANCE-FANCC CC Interactions

A FANCC-FANCE interaction and their association with other members of the CC have been documented by yeast and mammalian 2- and 3-hybrid assays, IVT studies and co-IPs [28, 45–47, 59, 62]. The central part of FANCE, residues 149–371, is required for the FANCC interaction as seen by yeast and mammalian 2-hybrid experiments [46, 47]. However, the corresponding regions of FANCC required for the interaction were not determined. More recent studies employing mammalian and yeast 3-hybrid assays indicate the importance of a FANCC-FANCE interaction to facilitate a direct interaction with FANCF [59, 61]. This interaction of FANCE with FANCF explains early co-IP findings of FANCEs associations with FANCA, FANCG, and FANCF [45, 62], as FANCF has been shown to directly interact with FANCG, and FANCG directly interacts with FANCA, indicating a possible indirect association of these proteins. 

### 3.4. FANCL-FANCB CC Interactions

Research has indicated FANCL is required to form a stable CC using co-IP experiments and size exclusion chromatography [63, 64]. Alpi et al. [64] show that in a wild-type chicken DT40 lymphoblastoid cell line, a complex of 1.5 MDa pulled out using a Tandem-affinity tagged FANCC exists. In corresponding FANCL-null cells, this complex is both less abundant and a lower molecular weight. However, the 1.5 MDa complex is established again upon expressing FANCL [64]. Consistent with these data, co-IPs in FANCL-null cells show a disruption of the interactions of FA CC proteins [63]. In the same study, a mammalian 2-hybrid assay indicates that FANCL forms a direct interaction with the CC via FANCB [63] (Figure 3). Medhurst et al. [63] also suggest that FANCL-FANCA interactions are mediated through FANCB and that FANCG is required to stabilize FANCA in this interaction. Additionally Alpi et al. [64] demonstrate that the stabilizing role of FANCL for the CC is independent from its E3 ligase activity, as point mutations that disrupt the RING domain and inhibit the monoubiquitination activity can still form a stable CC when introduced into the FANCL-null cell line. It is clear that FANCL is an important member of the CC for stability; however, molecular details, including the stoichiometry and domain requirements of how FANCL interacts with the CC are still lacking. 

The intricacy of, these CC protein-protein interactions is further complicated by the findings that a FANCA-FANCG interaction is required in stabilizing a FANCC-FANCA interaction and the need for FANCE to support the FANCA-FANCC interaction [25, 62]. 

The extensive research described here reflects a complex network of interactions between the CC proteins (summarized in Figure 3), all of which seem to be a requirement for a fully stable CC.

## 4. Subcomplexes, Stoichiometry, and Assembly of the CC

As discussed above FANCA has an NLS and monoubiquitinated FANCD2 locates at nuclear foci on chromatin as seen by fluorescent microscopy and co-IP studies [7, 65, 66]. However, FANCD2 has also been located in the cytoplasm [67–70] as have several of the CC components [8, 27, 51, 71]. Such findings give rise to the possibility that subcomplexes of the CC exist and localize in different cellular regions. 

Meetei et al. [72] reported different ratios of CC proteins observed in their co-IPs studies suggesting the idea of subcomplexes, although this could also reflect different stoichiometry of the CC proteins. An analysis of CC proteins isolated from the cytoplasm at different stages of the cell cycle revealed different molecular weight protein complexes by size exclusion chromatography [73]. A complex that consists of a single copy of each FA protein of the CC would give an approximate 737 kDa complex, which would increase to 861 kDa if FAAP24 and FAAP100 were included. Thomashevski et al. [73] report a 600 kDa cytoplasmic complex that increases to a 750 kDa complex during mitosis, supporting the idea of subcomplexes. One study documents such a subcomplex: FANCL-FANCB-FAAP100, which was tandem affinity purified from HeLa cell extracts [29]. Ling et al. [29] also suggest this subcomplex has a stoichiometric ratio of 1 : 1 : 1 in both cytoplasmic and nuclear extracts, with a more prominent association with FANCA in the nucleus. They speculate FANCA along with FANCM may localize the FANCL-FANCB-FAAP100 subcomplex to the nucleus [29]. Medhurst et al. [63] also support the idea of subcomplexes as they reveal from immunoprecipitation experiments of FANCG that FANCA and FANCL are coprecipitated independently of FANCE, FANCC, and FANCF. Studies identifying the localization of individual CC proteins by fluorescence microscopy in cells indicate FANCF and FANCE, which give a joint molecular weight of 101 kDa, are predominantly located in the nucleus, independently of any other CC proteins [27, 45, 47, 62]. FANCEs absence from any cytoplasmic complex described by Thomashevski et al. [73] further supports FANCEs localization in the nucleus. As a full complement of CC proteins is required for the monoubiquitination event, the observations of cytoplasmic subcomplexes and nuclear localization of certain CC proteins prompt the idea of the assembly of the full CC in the nucleus. 

The study by Thomashevski et al. [73] supports the idea of the full CC residing in the nucleus, as they report a large nuclear complex of 2 MDa and a 1 MDa chromatin associated complex, both containing CC proteins. As stated above, a CC consisting of one copy of each protein would give an approximate molecular weight of 861 kDa (including FAAP24 and FAAP100). Their reports suggest the nuclear and chromatin complexes may contain multiple copies of CC proteins and indicate the associations of the CC proteins and their stoichiometry differ between the two nuclear and chromatin complexes. Additionally components of these large nuclear complexes are likely to include other nuclear proteins, such as the BLM proteins and MHF1 and MHF2. Meetei et al. [72] report a 1.5–2 MDa complex when immunoprecipitating the BLM complex and found this complex to contain CC proteins. FANCM has since been appointed the CC protein interacting with the BLM proteins, as shown by co-IPs, *in vitro* translational work and fluorescence microscopy [74]. Likewise, Yan et al. [35] report a 1 MDa complex containing CC proteins and the FANCM-associated histone-fold proteins 1 and 2 (MHF1 and MHF2). Elucidating the existence of subcomplexes and a large nuclear CC certainly complicates the understanding of the CC. However, these studies highlight the importance of understanding both the assembly and stoichiometry of the CC and its subcomplexes. Whether there are additional roles for the subcomplexes is not yet understood. 

## 5. FANCM: A Member of the CC?

FANCM is considered a member of the CC, as it co-immunoprecipitates with other CC proteins and the loss of FANCM results in a loss of DNA damaged induced monoubiquitination and nuclear localization of other CC proteins [3, 30, 75, 76]. Indeed FANCM is thought to promote DNA damage-induced monoubiquitination of FANCD2 by recruitment of the CC via FANCF through its MM1 region [74]. Deans and West [74] also show deletions of regions throughout FANCF reduce an interaction with FANCM and deletion of FANCF residues 1–158 completely disrupt this interaction. The C-terminal end of FANCM associates with FAAP24 and both are thought to stabilize one another, [30, 76]. Ciccia et al. [30] also suggest the stability of FANCM-FAAP24 complex may be dependent on FANCB. However, Kim et al. [77] suggest FANCM recruits the CC proteins to chromatin and is not required for a stable CC. The histone-fold proteins MHF1 and MHF2 form another complex with FANCM [78] and are suggested to aid with the remodelling of DNA, as seen by co-IPs, size exclusion chromatography and DNA binding assays [34, 35]. Although the loss of MHF1 and MHF2 results in a loss of FANCD2 DNA damage inducible monoubiquitination, in agreement with Kim et al. [77], Yan et al. [35] report more than 70% of this complex is independent from the CC. A recent structural analysis reveals that MHF1 and MHF2 form a heterotetrameric complex and that disrupting the heterotetrameric interfaces results in an increased sensitivity to DNA damaging agents as seen by methyl methanesulfonate (MMS) treatment sensitivity assays in yeast [78]. A loss of FANCM has shown a loss of DNA damage inducible monoubiquitination of FANCD2 and many groups have suggested its role as a member of the CC; however, evidence is directing its role upstream of the CC suggesting it acts as a platform to recruit proteins to DNA. Additional evidence shows the FANCM-FAAP24 subcomplex has also been associated with interactions of the BLM complex [30, 74]. The role of FANCM in both Bloom syndrome and FA explains the similarities of the BLM and FA patients' high predisposition to cancer. Additionally the FANCM-FAAP24 subcomplex has also been implicated in ataxia telangiectasia and Rad3-related protein (ATR), a protein kinase associated with cell cycle arrest and checkpoint signalling independently from the rest of the CC proteins, through binding HCLK2 [79]. 

## 6. Discussion

The FA pathway has rapidly expanded over the last 15 years to a current count of 15 proteins. The number of FA proteins reflects the complicated nature of understanding the FA pathway, particularly the CC, which consists of over half of the FA proteins. Ascertaining functions for the FA proteins have been exceptionally challenging due to the lack of information divulged from the primary amino acid sequences. Extensive efforts have been made by researchers to define the roles of the individual CC proteins within the CC and to understand the need for such a large CC. However, there are still many remaining questions.

1. How does the CC support FANCLs E3 ligase activity? Is there a requirement for other CC proteins to localize FANCL to the nucleus? Or do the other CC proteins act as a structural scaffold for the monoubiquitination event?
2. Do the subcomplexes come together to form a full CC? If so how and where does the assembly take place? And are there independent roles for the subcomplexes?
3. What are the molecular and stoichiometric details of the CC? The requirement for all CC proteins for the monoubiquitination event is clear from patients with defects in the CC. Therefore, understanding the molecular details of the protein interactions that occur in the CC is key, because therapeutics could be designed to restore or diminish these interactions and furthermore tailored to the different FA complementation groups. The combination of biochemical, biophysical, clinical, and cell work will in time answer these questions.

## Figures and Tables

**Table 1:** Fanconi Anemia genes and their products.

Gene	MW (kDa)	No. of amino acids	Patient mutations
A	163	1455	60%
B	98	859	2%
C	63	558	13%
D1	384	3418	2%
D2	164	1451	3%
E	59	536	3%
F	42	374	3%
G	68	622	9%
I	150	1328	1%
J	141	1249	2%
L	42	375	0.2%
M	232	2048	0.2%
N	131	1186	0.6%
O	42	376	0.5%
P	200	1834	0.5%
FAAP24	24	215	—
FAAP100	100	881	—
MHF1	16	138	—
MHF2	10	81	—

Amino acids numbers were taken from the NCBI webserver, and patient mutational information was obtained from the Rockerfeller FA Mutations Database and was calculated as a percentage of all individuals recorded in the database.
